# Events associated with DNA replication disruption are not observed in hydrogen peroxide-treated *Escherichia coli*

**DOI:** 10.1093/g3journal/jkab044

**Published:** 2021-02-16

**Authors:** Chettar A Hoff, Sierra S Schmidt, Brandy J Hackert, Travis K Worley, Justin Courcelle, Charmain T Courcelle

**Affiliations:** Department of Biology, Portland State University, Portland, OR97201, USA

**Keywords:** RecF, glycosylases, DNA replication, DNA repair, manganese

## Abstract

UV irradiation induces pyrimidine dimers that block polymerases and disrupt the replisome. Restoring replication depends on the *recF* pathway proteins which process and maintain the replication fork DNA to allow the lesion to be repaired before replication resumes. Oxidative DNA lesions, such as those induced by hydrogen peroxide (H_2_O_2_), are often thought to require similar processing events, yet far less is known about how cells process oxidative damage during replication. Here we show that replication is not disrupted by H_2_O_2_-induced DNA damage *in vivo*. Following an initial inhibition, replication resumes in the absence of either lesion removal or RecF-processing. Restoring DNA synthesis depends on the presence of manganese in the medium, which we show is required for replication, but not repair to occur. The results demonstrate that replication is enzymatically inactivated, rather than physically disrupted by H_2_O_2_-induced DNA damage; indicate that inactivation is likely caused by oxidation of an iron-dependent replication or replication-associated protein that requires manganese to restore activity and synthesis; and address a long standing paradox as to why oxidative glycosylase mutants are defective in repair, yet not hypersensitive to H_2_O_2_. The oxygen-sensitive pausing may represent an adaptation that prevents replication from occurring under potentially lethal or mutagenic conditions.

## Introduction

All cells must accurately duplicate their genomes to reproduce. However, DNA damage can block the replication machinery and prevent it from completing this task, resulting in mutations if the wrong base is incorporated, rearrangements if replication resumes from the wrong site, or cell lethality if the block to replication cannot be overcome (reviewed in (Courcelle and Hanawalt 2003)). In order to understand how the cell maintains its genome, it is important to characterize how replication accurately processes DNA damage in these situations.

DNA damage generated by UV irradiation has often been used as a model to study how cells respond to and recover from DNA damage (Courcelle *et al.* 2003). Irradiation with 254-nm light induces pyrimidine dimers that block DNA polymerases and disrupt replisome progression (Setlow *et al.* 1963; Chan *et al.* 1985; Courcelle *et al.* 1997). We use the term disruption to distinguish it from replisome pausing or inactivation. These latter terms imply that the replisome remains structurally intact, and could simply resume once a missing precursor or cofactor is resupplied. In *Escherichia coli*, the disruption of replication at UV-induced lesions involves uncoupling of the leading and lagging strand, exposure of the nascent DNA to exonucleolytic degradation, and the dissociation of several components of the replisome, including Pol III, the beta clamp, and the tau complex, whereas both helicase and primase remain associated with the branched replication fork DNA (Courcelle and Hanawalt 1999; Pages and Fuchs 2003; Jeiranian *et al.* 2013). The integrity of the replication fork DNA and its fork structure remains intact throughout the recovery process, as no collapsed forks or broken linear DNA are observed (Courcelle *et al.*, 2003; Chow and Courcelle, 2004). Restoring replication and the replisome after disruption requires enzymatic processing by several RecF pathway gene products which recruit RecA and function to maintain and process the replication fork DNA so that the lesion can be repaired by nucleotide excision repair (Courcelle *et al.* 1997; Courcelle and Hanawalt, 1999; Courcelle *et al.* 1999, 2003; Chow and Courcelle 2004; Courcelle *et al.* 2006; Bichara *et al.* 2007). In the absence of enzymatic processing by RecF, the DNA at the disrupted fork is extensively degraded and replication fails to resume (Courcelle *et al.* 1997, 1999, 2003). The recovery of replication is also heavily dependent on the removal of the UV lesions by the UvrABC excinuclease complex which incises a 12-bp region surrounding the damaged bases (Setlow *et al.* 1963; Courcelle *et al*, 1997, 1999). UvrD helicase, DNA polymerase I, and ligase then remove, resynthesize, and join the missing bases to complete repair (Caron *et al.* 1985; Van Houten 1990). In *uvrA, uvrB*, or *uvrC* mutants, these lesions are not removed, the replication forks remain blocked, and the resumption of DNA replication is severely impaired (Setlow *et al.* 1963; Howard-Flanders *et al.* 1969; Courcelle *et al.* 1999, 2003). In these repair-defective cells, high frequencies of chromosomal exchanges and extensive cell death are observed (Howard-Flanders and Theriot 1966; Rupp and Howard-Flanders 1968; Rupp *et al.* 1971; Courcelle and Hanawalt 2001; Courcelle *et al.* 2001; Bichara *et al.* 2007). In repair-proficient cells, these recombination events are efficiently suppressed, survival is greatly enhanced, and a robust recovery of replication is observed, indicating that the normal mechanism of recovery is integrated with lesion repair (Howard-Flanders *et al.* 1969; Rupp *et al.* 1971; Courcelle and Hanawalt 2001; Courcelle *et al.* 2001; Bichara *et al.* 2007).

In addition to UV, other environmental agents interact with and damage DNA, including ionizing radiation and a variety of chemicals (Singer and Kusmierek 1982; Hutchinson 1985). Of particular importance is the damage generated by both endogenous and exogenous sources of reactive oxygen species. DNA damage induced by reactive oxygen species is thought to be a primary source of the mutations in the etiology of cancer and aging in humans and is used as defense by our immune systems to kill invading pathogens (Fridovich 1978; Ames 1983; Cross *et al.* 1987; Nathan and Cunningham-Bussel 2013). Reactive oxygen species, such as that formed by H_2_O_2_ treatment, induce a broad spectrum of distinct base modifications in DNA (reviewed in (Wallace 2002)). These include lesions that have been characterized *in vitro* to maintain their coding specificities, such as dihydrothymine; that mispair during replication or transcription, such as 8-oxo-guanine; and others that block some DNA and RNA polymerases and are potentially lethal, such as thymine glycol (Ide *et al.* 1991; Evans *et al.* 1993; Efrati *et al.* 1999). Similar to UV damage, oxidized base damage is also repaired by excision and resynthesis of damaged bases. However, in this case, the excision is catalyzed by a lesion-specific DNA glycosylase that cleaves the glycosidic bond and releases the damaged bases. Most oxidative glycosylases also have an associated AP-lyase activity that incises the sugar-phosphate backbone (Bailly and Verly 1987; Melamede *et al.* 1994; Bhagwat and Gerlt 1996; Jiang *et al.* 1997). The apurinic or apyrimidinic (AP) site is then either incised or processed by an AP endonuclease to generate a clean 3′-OH that can be extended and joined by DNA polymerase I and ligase to restore the template’s integrity (reviewed in (Wallace, 1994)).

Little is known about how replication recovers after DNA damage induced by oxygen free radical species. It is often assumed that the processing and recovery of replication in the presence of oxidative DNA damage will mimic those observed in the presence of UV-induced damage. However, mutants lacking the DNA glycosylases responsible for repairing oxidized-base damage are as resistant as wild-type cells when treated with H_2_O_2_ (Laspia and Wallace 1988; Asad *et al.* 1995; Saito *et al.* 1997; Schalow *et al.* 2011). Similarly, mutants deficient in nucleotide excision repair are also resistant to H_2_O_2_ (Imlay and Linn 1987; Asad *et al.* 1995; Schalow *et al.* 2011). Recently, we also observed that the recovery of replication after oxidative challenge had a unique requirement for manganese, something that is not observed after UV-induced damage (Hutfilz *et al.* 2019). These observations raise the possibility that the mechanism by which replication deals with H_2_O_2_-induced DNA damage is distinct from that seen after UV. Here we considered this possibility, and found that oxidative lesions induced by H_2_O_2_ do not disrupt replication, and that the resumption of DNA synthesis does not require any enzymatic processing by RecF, implying that the replisome remains intact but is simply inactive due to the lack of metal cofactors.

## Materials and methods

###  

#### Bacterial strains and plasmids

SR108 (*thyA36 deoC2* derivative of W3110), HL921 (SR108 Δ(*srlR-recA*)*306*::Tn*10*), HL922 (SR108 *recB21recC22 argA81*::Tn*10*), HL952 (SR108 *uvrA*::Tn*10*), CL008 (SR108 *recG258*::Tn*5*), CL544 (SR108 *recR6212*::*cat*), CL577 (SR108 *ruvC53eda-51*::Tn*10*), CL579 (SR108 *recF6206*::tet^R^), and CL915 (SR108 *recN*::*cat*) have been previously described (Mellon and Hanawalt 1989; Courcelle *et al.* 1997, 1999, 2003; Donaldson *et al.* 2004, 2006; Cole *et al.* 2018). CL1746 (SR108 *nth*::kan^R^ *nei*::*cat*) was constructed by P1 transduction of the *nei*::*cat* allele from CL1005 (SR108 *nei*::*cat* (Schalow *et al.* 2011)) into CL1006 (SR108 *nth*::kan^R^ (Schalow *et al.* 2011)). CL1941 (SR108 *nth*::kan^R^ *nei*::*cat fpg*::tet^R^) was constructed by P1 transduction of the *fpg*::tet^R^ allele from CL1778 (SR108 *fpg*::tet^R^ (Schalow *et al.* 2011)) into CL1746. CL1155 (DY329 *xthA*::*cat*) was constructed by gene replacement using the recombineering strain DY329 (Yu *et al.* 2000). The *cat* cassette from pPCR-Script Cam was amplified using the primers 5′GTCTCTTTTAATATCAACGGCCTGCGCGCCAGACCTCACTGTGACGGAAGATCACTTCG and 5′CGGTTTTTCCATGCTGCGGATTTCATAGTCGATGCCGGTACCAGCAATAGACATAAGCG. The PCR product was transformed into DY329 to generate CL1155, selecting for chloramphenicol resistance. The gene replacement was then moved into SR108 by standard P1 transduction to generate CL1168 (SR108 *xthA*::*cat*). All strains used in this work are summarized in Supplementary Table S1.

pBR322 is a medium copy number, ColE1-based, 4.4-kb plasmid (Promega).

#### H_2_O_2_ survival assays

Fresh overnight cultures were diluted 1:100 in Davis medium supplemented with 0.4% glucose, 0.2% casamino acids and 10 µg/ml thymine (DGCthy), grown at 37°C to an OD_600_ of 0.3, and then treated with 10 mM H_2_O_2_. At the times indicated, 0.1-ml aliquots of each culture were removed and serially diluted in 10-fold increments into DGCthy medium. Triplicate 10-µl aliquots of each dilution were then spotted on Luria-Bertani (LB) plates supplemented with 10 µg/ml thymine (LBthy). Viable colonies were counted following overnight incubation at 37°C.

#### UV survival assays

UV irradiation used a 15-W germicidal lamp (254 nm) at an incident dose of 0.9 J/m^2^/s. Fresh overnight cultures were diluted 1:100 in DGCthy medium and grown at 37°C to an OD_600_ of 0.3. At this time, 0.1-ml aliquots of each culture were removed and serially diluted in 10-fold increments into DGCthy medium. Triplicate 10-µl aliquots of each dilution were then spotted on LBthy plates and irradiated with increasing doses of UV as indicated. Viable colonies were counted following overnight incubation at 37°C.

#### Lesion frequency

For UV irradiation, fresh overnight cultures were diluted 1:100 and grown at 37°C in DGCthy medium to an OD_600_ of 0.3. Where indicated 200 µM manganese (II) chloride (MnCl_2_·4H_2_O) was added to the medium as well. At this time, cultures were irradiated with an incident dose of 50 J/m^2^ and then returned to 37°C to allow recovery. For H_2_O_2_ challenge, fresh overnight cultures were diluted 1:100 in DGCthy medium supplemented with MnCl_2_ as indicated, grown at 37°C to an OD_600_ of 0.3, and then treated with 10 mM H_2_O_2_ for 5 min at 37°C. Cells were filtered on 0.45-µm membranes (Fisherbrand) to remove excess H_2_O_2_ from the medium, resuspended in fresh DGCthy medium supplemented with manganese as appropriate to initial growth conditions and returned to 37°C for the duration of the time course. For both treatments, a 0.75-ml aliquot was transferred at the times indicated to an equal volume of 2× 200 mM NaCl, 20 mM Tris, pH 8.0, 40 mM EDTA, pH 8.0 (NET). Cells were pelleted and resuspended in 0.14-ml of lysis buffer (1 mg/ml lysozyme, 0.5 mg/ml RNaseA in 10 mM Tris, pH 8.0, 1 mM EDTA, pH 8.0) and incubated for 30 min at 37°C. Then, 0.01-ml 10 mg/ml ProteinaseK and 0.01-ml 20% Sarkosyl were added to the samples and incubation was continued for 30 min at 37°C. The samples were then extracted once with four volumes of phenol:chloroform, followed by two volumes of chloroform, and dialyzed against 200-ml 1 mM Tris, pH 8.0, 1 mM EDTA, pH 8.0 for 30 min using 47-mm Millipore 0.025-µm pore disks.

For UV-irradiated samples, 15 µl of each DNA sample was treated with reaction buffer (12.5 mM sodium phosphate [pH 6.8], 5 mM EDTA [pH 8.0], 50 mM NaCl, 0.5 mM dithiothreitol, 0.005% Triton X-100, 0.1 mg/m bovine serum albumin) supplemented with either no enzyme or 2 U T4 endonuclease V (T4 Endo V; Trevigen) for 1 h at 37°C. For H_2_O_2_-treated samples, 15 µl of each DNA sample was treated with reaction buffer (30 mM EDTA [pH 8.0], 22.5 mM NaCl, 5 mM Tris [pH 8.0]) supplemented with either no enzyme or 0.53 µM Fpg glycosylase and 2.7 µM Endo III for 1 h at 37°C. Enzyme preparations were titrated using purified undamaged genomic DNA as a template. The highest enzyme concentration that did not exhibit nonspecific activity on undamaged DNA was used. For the preparations in our lab, this corresponded to 2 U T4 Endo V, 0.53 µM Fpg glycosylase and 2.7 µM Endo III. Treated samples were then electrophoresed on a 0.5% alkaline agarose gel in 30 mM NaOH, 1 mM EDTA at 30 V for 16 h, stained and visualized with ethidium bromide. The intensity of each high-molecular-weight band was determined using ImageQuant software (GE Biosciences). The fraction of lesion-free DNA fragments was quantified as a ratio of high-molecular-weight DNA in the T4 Endo V- or Fpg/Endo III-treated band (Enz_T_) to the band without enzyme treatment (NoEnz_T_) at each time point, T. To normalize for any nicks or AP sites present in the DNA before UV or H_2_O_2_ treatment, the ratio obtained at each time point was divided by the ratio calculated at the pretreatment time point as follows: (Enz_T_/NoEnz_T_)/*R*_0_, where *R*_0_ is (Enz_T_/NoEnz_T_) at the time immediately before UV or H_2_O_2_ addition.

#### DNA synthesis rate

For experiments using UV irradiation, overnight cultures were diluted 1:100 and grown at 37°C in DGCthy to an OD_600_ of 0.25–0.35. Where indicated manganese was added to the medium as described above. At this time, half of the cells were mock irradiated, while the other half of the culture was irradiated with 50 J/m^2^.

For experiments using H_2_O_2_, overnight cultures were diluted 1:100 and grown at 37°C to an OD_600_ of 0.25–0.35 in DGCthy supplemented with 200 µM MnCl_2_ where indicated. At this time, half of the cells were mock treated, while the remaining culture was exposed to 10 mM H_2_O_2_ for 5 min at 37°C. Following either mock or H_2_O_2_ treatment, cells were filtered on 0.45-µm membranes to remove excess H_2_O_2_ from the medium and resuspended in fresh DGCthy medium either supplemented with Mn or not based on initial growth conditions.

For both UV irradiation and H_2_O_2_ experiments, cultures were returned immediately to 37°C after treatment to allow recovery and continued growth. At the times indicated, duplicate 0.5-ml aliquots of culture were pulse-labeled with 0.5 µCi/ml [^3^H]thymidine for 2 min at 37°C. Cells were then lysed, and the DNA was precipitated in cold 5% trichloroacetic acid and filtered onto Millipore glass fiber filters. The amounts of ^3^H on each filter were determined by scintillation counting.

#### Two-dimensional agarose gel electrophoresis

Cells containing the plasmid pBR322 were grown overnight in DGCthy medium supplemented with 100 µg/ml ampicillin (amp). A 0.2-ml aliquot of this culture was pelleted and resuspended in 20-ml DGCthy with 200 µM MnCl_2_ where indicated and grown without amp selection to OD_600_ of 0.5. At this time, cultures were either UV-irradiated with an incident dose of 50 J/m^2^ and then returned to 37°C to allow recovery, or treated with 10 mM H_2_O_2_ for 5 min at 37°C, filtered on 0.45-µm membranes to remove excess H_2_O_2_ from the medium, then resuspended in fresh DGCthy medium and returned to 37°C for the duration of the time course. At the times indicated, a 0.75-ml aliquot was transferred to an equal volume of 2× NET. Total DNA (genomic and plasmid) was then purified from these cultures as described above.

DNA samples were dialyzed against 200-ml 1 mM Tris, pH 8.0, 1 mM EDTA, pH 8.0 for 30 min using 47-mm Millipore 0.025-µm pore disks and digested with PvuII restriction endonuclease (Thermo Fisher Scientific) overnight at 37°C. Samples were then extracted once with one volume of chloroform, loaded directly on a 0.4% agarose gel in 1× TBE (Tris-borate-EDTA) and DNA was separated initially at 1 V/cm for 15 h. For the second dimension, lanes were excised, rotated 90°, recast in 1% agarose in 1× TBE and electrophoresed at 6.5 V/cm for 7 h. DNA in the gel was transferred to Hybond N+ nylon membrane by standard Southern blotting techniques, and the plasmid DNA was detected using ^32^P-labeled pBR322 plasmid prepared by random prime labeling (Agilent) using dCTP (3000 Ci/mmol; PerkinElmer) and visualized using a Storm 840 PhosphorImager (GE Biosciences) and its associated ImageQuant software.

## Data availability

Strains used in these experiments are available upon request and can be found in Supplementary Table S1. Supplemental material is available at figshare: https://doi.org/10.25387/g3.13426079.

## Results

### DNA replication recovers in the absence of lesion removal following oxidative challenge

**Figure 1:**
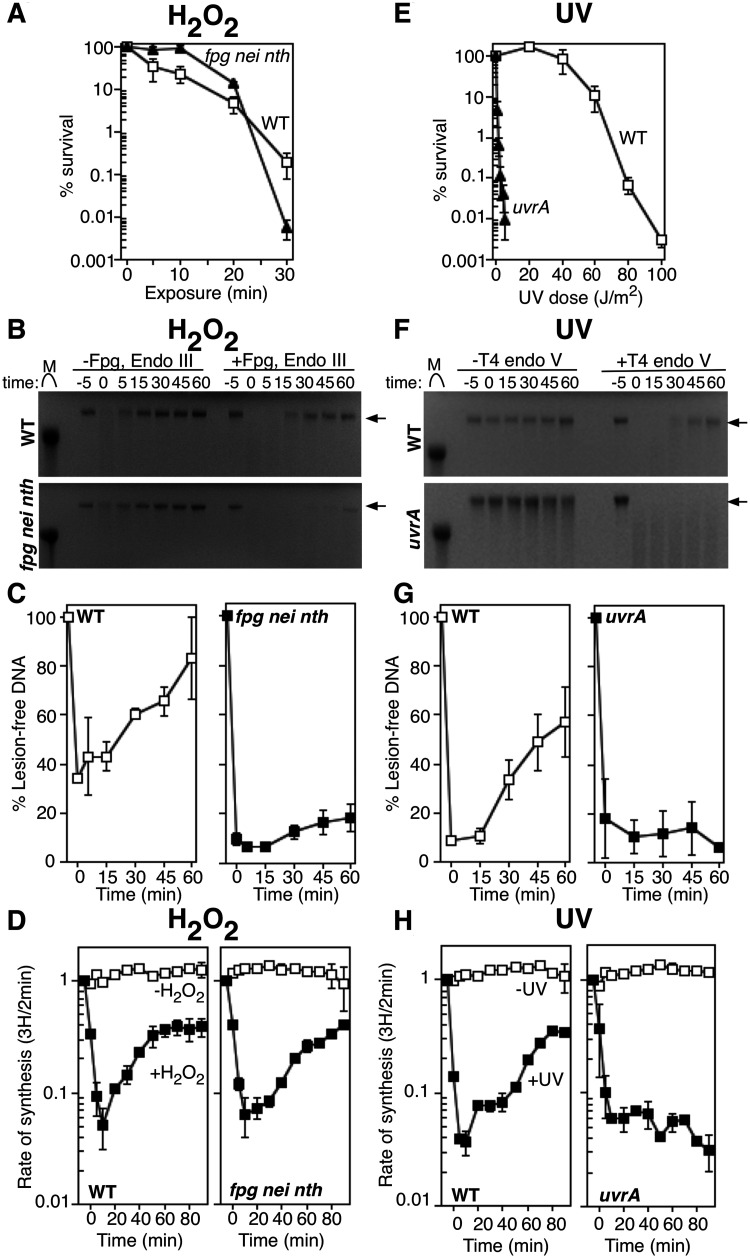
DNA replication recovers even in the absence of lesion removal following H_2_O_2_ treatment, but not UV irradiation. (A) The survival of wild-type (open squares) and *fpg nei nth* (filled triangles) cells following 10 mM H_2_O_2_ treatment is plotted. Graphs represent the average of three independent experiments. Error bars represent one standard error of the mean. (B) Wild-type (top) and *fpg nei nth* (bottom) cultures were treated with 10 mM H_2_O_2_ for 5 min, allowed to recover and then genomic DNA was purified at the indicated times. DNA was either treated with Fpg and Endo III (+Fpg, Endo III), or no glycosylase (untreated) for 1 h at 37°C and then analyzed on alkali agarose gels. A representative gel is shown. Arrows indicate lesion-free DNA. (C) The percentage of lesion-free, high-molecular-weight DNA in Fpg/Endo III-treated samples is plotted for each time point relative to mock-treated samples. Graphs represent an average of at least two independent experiments. (D) [^3^H]thymidine was added to cultures grown with manganese for 2 min at the indicated times following H_2_O_2_ treatment at time zero. The rate of DNA synthesis (^3^H/2 min) relative to the amount incorporated immediately prior to exposure is plotted for wild-type and *fpg nei nth* cultures exposed to mock treatment (open symbols) or 10 mM H_2_O_2_ for 5 min (filled symbols). Graphs represent an average of at least two independent experiments. Error bars represent one standard error of the mean. (E) The survival of wild-type (open squares) and *uvrA* (filled triangles) cells following UV irradiation is plotted. Graphs represent the average of three independent experiments. Error bars represent one standard error of the mean. (F) Wild-type (top) and *uvrA* (bottom) cultures were UV irradiated at 50 J/m^2^, then genomic DNA was purified at the indicated times and either treated with T4 endonuclease V (+T4 endo V) or no T4 endo V (untreated) for 1 h at 37°C and analyzed on alkali agarose gels. A representative gel is shown. Arrows indicate lesion-free DNA. (G) The percentage of lesion-free, high-molecular-weight DNA in T4 Endo V-treated samples is plotted for each time point relative to mock-treated samples. Graphs represent an average of at least two independent experiments. H) [^3^H]thymidine was added to cultures grown with manganese for 2 min at the indicated times following UV irradiation at time zero. The rate of DNA synthesis (^3^H/2 min) relative to the amount incorporated immediately prior to exposure is plotted for wild-type and *uvrA* cells exposed to mock treatment (open symbols) or 50 J/m^2^ UV irradiation (filled symbols) treatment. Graphs represent an average of at least two independent experiments. Error bars represent one standard error of the mean.

To examine how replication responds to H_2_O_2_-induced DNA damage *in vivo*, we compared the replication occurring in H_2_O_2_-treated wild-type cells to that occurring in *fpg nei nth* mutants, which lack the three predominant oxidative DNA glycosylases. Consistent with previous reports (Laspia and Wallace 1988; Asad *et al.* 1995; Saito *et al.* 1997; Schalow *et al.* 2011), the absence of Fpg, Endonuclease III (Endo III) and Endonuclease VIII (Endo VIII) did not render cells hypersensitive to H_2_O_2_ (Figure 1A). However, the absence of these glycosylases impaired or prevented the removal of the oxidative lesions recognized by these enzymes *in vivo* (Figure 1, B and C). To monitor repair, cultures were exposed to 10 mM H_2_O_2_ for 5 min, then filtered and resuspended in fresh medium to allow recovery in the absence of H_2_O_2_. Lesion removal was assayed at various times during the recovery by treating purified total genomic DNA with Fpg and Endo III (Schalow *et al.* 2011). Purified Endo VIII was not utilized because it lacks enzymatic turnover *in vitro* and its substrate specificity overlaps with Fpg and Endo III (Melamede *et al.* 1994; Jiang *et al.* 1997). The glycosylase activity of Fpg and Endo III results in incisions at lesions recognized by the three oxidative glycosylases, while the AP lyase activity associated with these enzymes nicks the DNA backbone (Bailly and Verly 1987; Melamede *et al.* 1994; Bhagwat and Gerlt 1996; Jiang *et al.* 1997). Any non-incised AP sites created by base excision would also be cleaved under the alkali conditions used during electrophoresis. Thus, following alkali-agarose gel electrophoresis, the presence of oxidative lesions in the genomic DNA is observed as the loss of high-molecular-weight species. In wild-type cells, prior to treatment, DNA fragments averaged greater than 40 kb in length, which is the approximate limit of resolution in our agarose gels. Incubation of the genomic DNA with Fpg and Endo III glycosylases resulted in a loss of high-molecular-weight fragments at times immediately after H_2_O_2_ exposure, indicating the presence of lesions. Over time, the number of lesions in the DNA decreased and within 30 min, greater than 60% of the DNA had been restored as lesion-free high-molecular-weight fragments (Figure 1C). In contrast, the removal of lesions in H_2_O_2_-treated *fpg nei nth* cultures was severely impaired with only 18% of high-molecular-weight DNA restored by the end of the 60-min recovery time course (Figure 1C). The actual amount of repair is likely to be significantly less as much of this high-molecular-weight DNA is likely to represent newly synthesized DNA, rather than repair events (see Figure 1D and text below).

To examine how H_2_O_2_-induced damage affects DNA replication, cultures were treated with 10 mM H_2_O_2_ for 5 min, as before, and at the times indicated, aliquots of cultures were pulse-labeled with [^3^H]thymidine for 2 min before the DNA was precipitated and the amount of ^3^H incorporated was quantified. The rate of DNA replication (^3^H incorporation in the DNA/2 min) was determined for each time point and expressed relative to the rate immediately prior to treatment. In both wild-type and *fpg nei nth* cells, a rapid inhibition of DNA replication was observed immediately following H_2_O_2_ treatment. Surprisingly, replication rapidly resumed even in the absence of lesion removal and the *fpg nei nth* mutant restored DNA synthesis with kinetics that were modestly altered or reduced relative to wild-type cells (Figure 1D). The result indicates that the resumption of replication following H_2_O_2_ treatment does not require lesion repair by the glycosylases tested.

The recovery of replication in the absence of oxidative lesion repair stands in contrast to what occurs after UV irradiation. *uvrA* mutants are defective for nucleotide excision repair and are unable to remove UV-induced lesions (reviewed in (Van Houten 1990)). Immediately after treatment with 50 J/m^2^ UV light, a similar inhibition of replication is observed in both wild-type and *uvrA* cultures. Using the pyrimidine dimer-specific glycosylase, T4 Endonuclease V (T4 Endo V), to monitor repair (Spivak and Hanawalt 1995) and measuring the rate of replication as before demonstrates that in wild-type cultures, replication resumes at a time that correlates with the removal of the lesions. However, in *uvrA* mutants, the lesions are not removed, DNA synthesis does not resume, and high levels of lethality are observed (Figure 1, E–H).

Thus, unlike UV-induced damage, the results demonstrate that lesions removed by Fpg, Endo III, or Endo VIII do not prevent replication from progressing *in vivo*, and imply that these lesions do not disrupt replication. However, an inhibition of replication is clearly observed immediately following exposure to H_2_O_2_. This latter observation would appear to suggest that replication is disrupted by these lesions. We considered two possibilities to explain these apparently contradictory observations. It is possible that other forms of H_2_O_2_-induced DNA damage, not recognized by these glycosylases, disrupt replication and are responsible for the inhibition that is observed. Alternatively, the inhibition could result from an oxidative sensor or another aspect of the cellular oxidative stress response that is not directly associated with DNA damage.

### The recovery of replication following H_2_O_2_ occurs independently of *recF* pathway and lacks intermediates associated with disrupted replication forks

**Figure 2:**
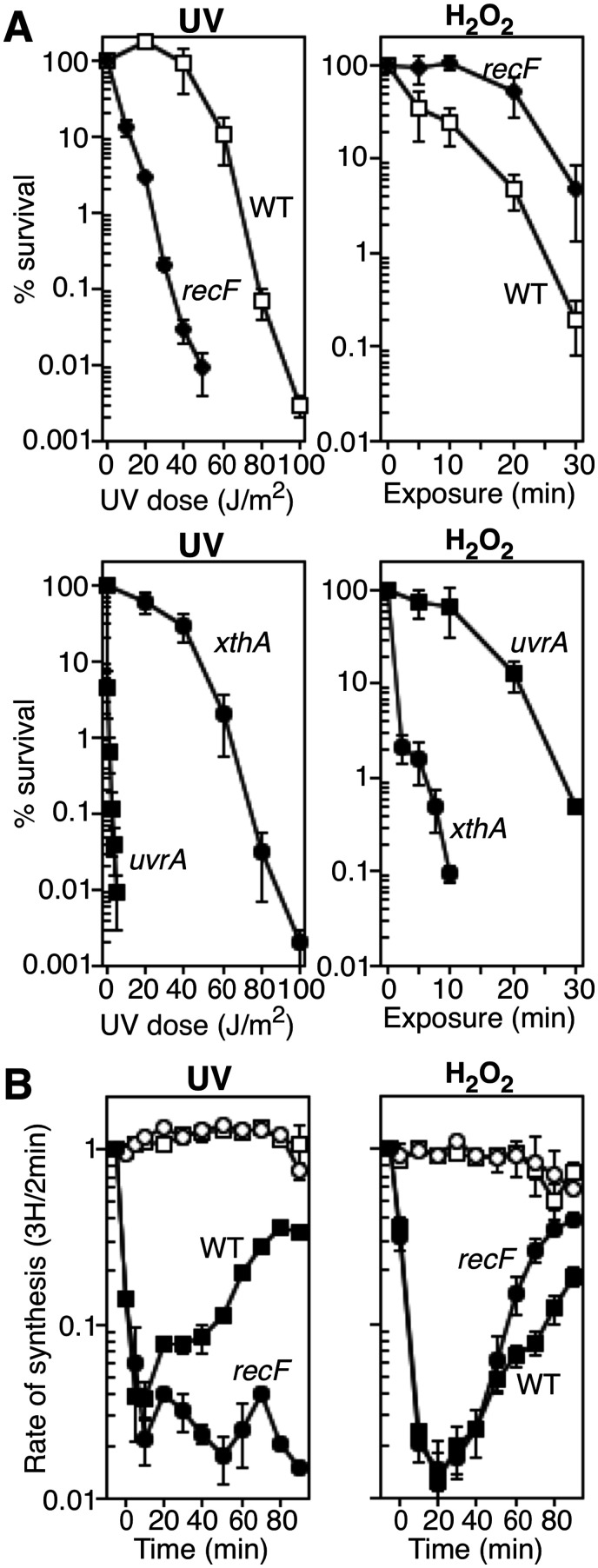
RecF is not required for survival or replication recovery following oxidative challenge. (A) The survival of wild-type (open squares), *recF* (filled diamonds), *uvrA* (filled squares) and *xthA* (filled circles) cells following UV irradiation or 10 mM H_2_O_2_ treatment is plotted. Wild-type plots for each treatment and *uvrA* plot for UV irradiation are reproduced from Figure 1 for comparison. Graphs represent the average of three independent experiments. Error bars represent one standard error of the mean. (B) Data were obtained and plotted as for Figure 1. The rate of DNA synthesis (^3^H/2 min) relative to the amount incorporated immediately prior to exposure is shown for wild-type and *recF* cells exposed to mock treatment (open symbols), UV irradiation, or 10 mM H_2_O_2_ exposure for 5 min (filled symbols) in Mn-supplemented medium. Graphs represent an average of at least two independent experiments. Error bars represent one standard error of the mean.

To determine whether the observed replication inhibition results from disruption by other forms of damage that are not repaired by the three predominant oxidative DNA glycosylases, we examined *recF* mutants. In contrast to other DNA damage hypersensitive mutants, the defect in *recF* pathway mutants is specific to their ability to restore replication following disruption (Supplementary Figures S1 and S2). Other hypersensitive mutants, including *recBCD*, *recG*, and *ruvABC* process and restore replication normally following disruption, arguing that cell lethality in these mutants arises from other causes (Courcelle *et al.* 1997; Courcelle and Hanawalt 1999; Courcelle *et al.* 2003; Chow and Courcelle 2004; Donaldson *et al.* 2004; Donaldson *et al.* 2006; Wendel *et al.* 2014). RecF processes and maintains DNA at replication forks following disruption by DNA damage, and is required for DNA synthesis to resume (Horii and Clark 1973; Courcelle *et al.* 1997; Courcelle *et al.* 1999; Courcelle *et al.* 2003). As shown in Figure 2A, we found that although RecF contributes to survival following UV irradiation, it does not contribute to survival following H_2_O_2_ exposure. Indeed, the absence of RecF conferred a modest resistance in the presence of H_2_O_2_ compared to wild-type cells. As controls, we examined *uvrA* and *xthA* (encoding the dominant AP endonuclease, Exo III) mutants (Figure 2A), which have previously been shown to be hypersensitive to UV and H_2_O_2_, respectively (Hill 1958; Setlow *et al.* 1963; Demple *et al.* 1983).

We also examined whether the absence of RecF impaired the recovery of DNA synthesis following exposure to H_2_O_2_, as before. Although RecF was required for replication to resume after UV irradiation (Figure 2B and (Courcelle *et al.* 1997, 1999)), DNA synthesis resumed independently of RecF following H_2_O_2_ treatment. Taken together, these results are consistent with the idea that the inhibition of replication caused by H_2_O_2_ is not due to disruption of replication by DNA damage, and demonstrate that RecF-mediated processing is not required for survival or for replication to resume.

**Figure 3:**
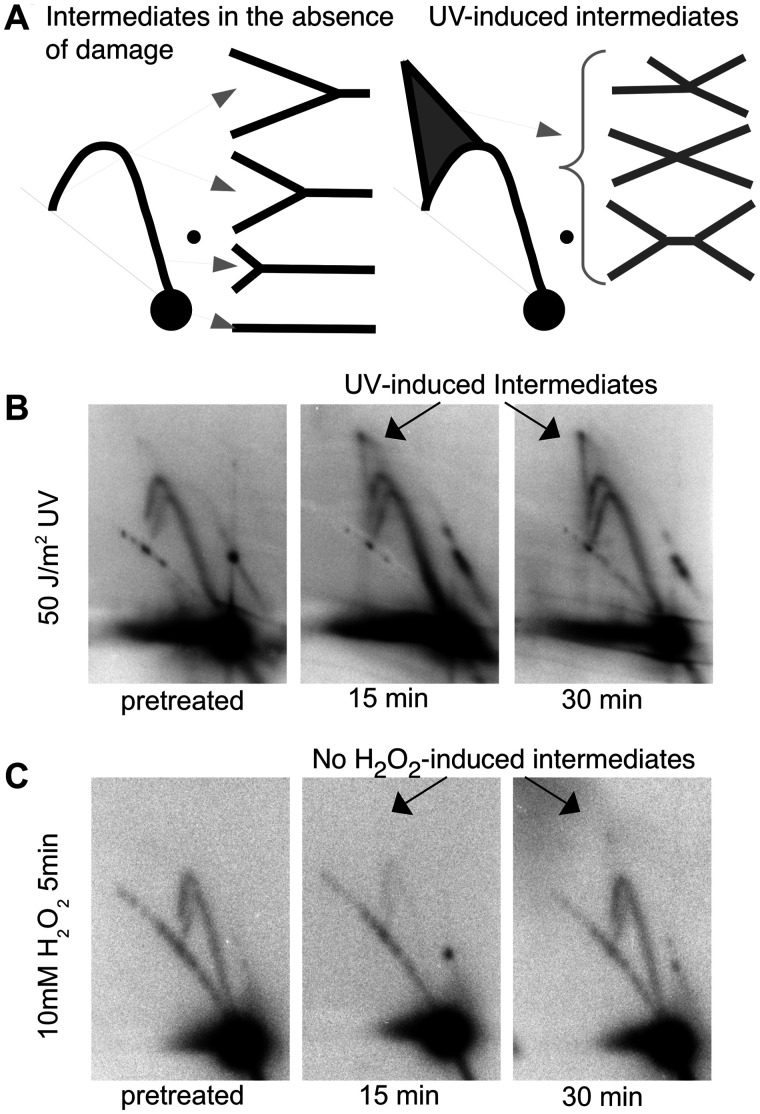
The inhibition, and subsequent recovery, of DNA replication after oxidative stress is not associated with DNA damage processing intermediates. (A) The migration pattern of PvuII-digested pBR322 plasmid DNA observed by 2D gel electrophoresis is diagrammed. Non-replicating plasmids run as a 4.4-kb linear fragment. Replicating plasmids form Y-shaped structures that migrate slower than non-replicating linear DNA and form an arc that extends from the linear region. Following UV irradiation, double-Y or X-shaped intermediates are observed that migrate in the cone region behind the arc of Y-shaped molecules. Two-dimensional agarose gels of total DNA purified from wild-type cultures containing plasmid exposed to (B) 50 J/m^2^ UV irradiation or (C) 10 mM H_2_O_2_ for 5 min were probed with labeled pBR322 at the indicated times after treatment. Arrow indicates double-Y or X-shaped intermediates in the cone region.

Previous studies have shown that replication forks disrupted by UV-induced DNA damage undergo processing events that displace the DNA polymerase and restore the region to its double-stranded form, allowing repair enzymes to access the lesion and effect repair (Courcelle *et al.* 2003; Chow and Courcelle 2004; Donaldson *et al.* 2006; Belle *et al.* 2007; Jeiranian *et al.* 2012). The processing forms unique structural intermediates that can be observed using 2D agarose gel electrophoresis. To determine if similar structural intermediates or processing occurs following the inhibition of replication observed after H_2_O_2_ stress, we compared the structural intermediates that arise on replicating plasmids after treatment with H_2_O_2_ to that occurring after UV irradiation using 2D agarose gel electrophoresis. To this end, wild-type cultures of *E. coli* containing the plasmid pBR322 were either exposed to H_2_O_2_ or irradiated with UV, as before. At various times following treatment, total genomic DNA was isolated, digested with the restriction enzyme, PvuII, to linearize the plasmid just downstream of its origin of replication, then separated and analyzed by 2D agarose gel electrophoresis. Non-replicating plasmid molecules migrate as a linear 4.4-kb fragment and form the prominent spot observed on the gels. Replicating molecules form Y-shaped structures that migrate more slowly due to their larger size and nonlinear shape, and appear as an arc radiating out from the prominent linear spot (Figure 3, A and B). Following the disruption of replication by UV-induced damage, the displacement of the DNA polymerase and processing of the replication fork DNA form a set of molecules that contain four arms (X- or double Y-shaped structures) which migrate slower through the gel and accumulate in the cone region located above the Y-arc (Figure 3, A and B). Previous work from our lab has shown that these cone region intermediates persist until the time corresponding with when the lesions are repaired and DNA replication resumes (Courcelle *et al.* 2003). When we examined H_2_O_2_-treated wild-type cells using this technique, we did not observe the accumulation of any cone region intermediates, despite the inhibition of replication (Figure 3C). Instead, only normal Y-shaped intermediates were observed throughout the recovery time course. The absence of any arrested replication fork intermediates indicates that H_2_O_2_-induced lesions are processed differently from UV lesions and suggests that following H_2_O_2_-treatment the replication fork machinery is not disrupted and the replication forks do not require processing similar to that which occurs after UV.

### DNA replication, but not DNA repair, contains an iron-sensitive component that is inactivated and restored by manganese after H_2_O_2_ treatment

**Figure 4:**
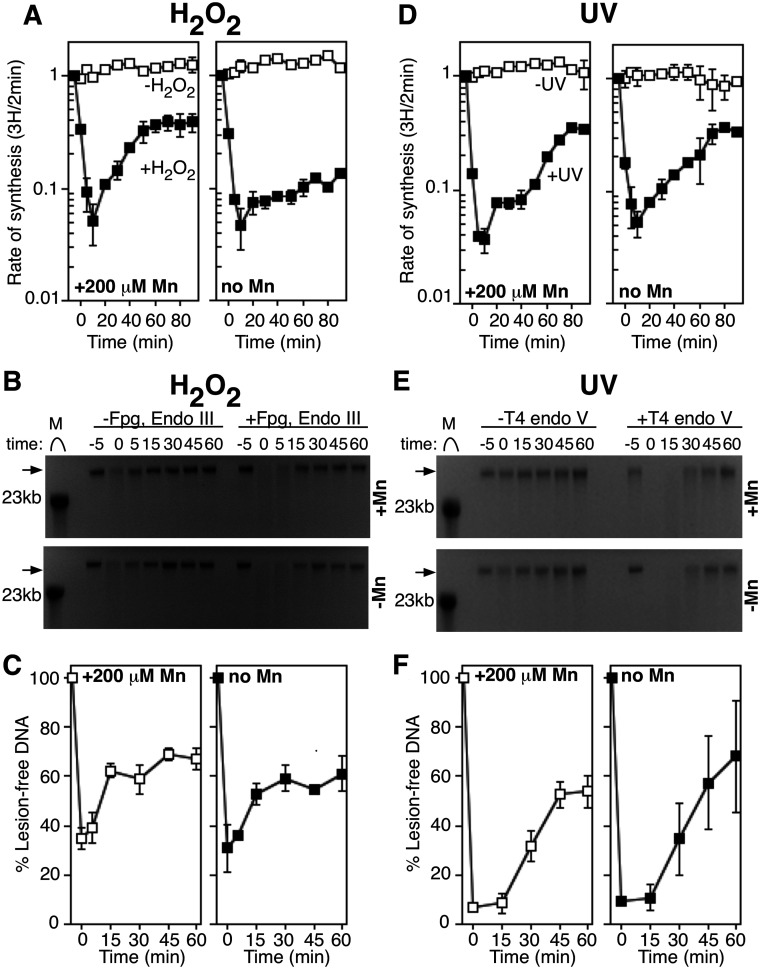
The absence of Mn in growth medium inhibits replication recovery, but not DNA repair, following oxidative stress. (A) Data were obtained and plotted as for Figure 1. The rate of DNA synthesis (^3^H/2 min) relative to the amount incorporated immediately prior to exposure is plotted for wild-type cells exposed to mock treatment (open symbols) or 10 mM H_2_O_2_ (filled symbols) treatment in the presence or absence of Mn. Mn-supplemented plot is reproduced from Figure 1C for the purpose of comparison and control. Graphs represent an average of at least two independent experiments. Error bars represent one standard error of the mean. (B) Wild-type cultures grown with (top) or without Mn (bottom) were treated with 10 mM H_2_O_2_ for 5 min, allowed to recover and then genomic DNA was purified at the indicated times. DNA was either treated with Fpg and Endo III (+Fpg, Endo III), or no glycosylase (untreated) for 1 h at 37°C and then analyzed on alkali agarose gels. A representative gel is shown for each treatment. Arrows indicate lesion-free DNA. (C) The percentage of lesion-free, high-molecular-weight DNA in Fpg/Endo III-treated samples is plotted for each time point relative to mock-treated samples. Graphs represent an average of at least two independent experiments. Error bars represent one standard error of the mean. (D) The rate of DNA synthesis (^3^H/2 min) relative to the amount incorporated immediately prior to exposure is plotted for wild-type cells exposed to mock treatment (open symbols) or 50 J/m^2^ UV irradiation (filled symbols) in the presence or absence of Mn. Mn-supplemented plot is reproduced from Figure 1. Graphs represent an average of at least two independent experiments. Error bars represent one standard error of the mean. (E) Wild-type cultures grown with (top) or without Mn (bottom) were UV irradiated at 50 J/m^2^, then genomic DNA was purified at the indicated times and either treated with T4 endo V (+T4 Endo V) or no T4 endo V (untreated) for 1 h at 37°C and analyzed on alkali agarose gels. A representative gel is shown for each treatment. Arrows indicate lesion-free DNA. (F) The percentage of lesion-free, high-molecular-weight DNA in T4 Endo V-treated samples is plotted for each time point relative to mock-treated samples. Graphs represent an average of at least two independent experiments. Error bars represent one standard error of the mean.

In previous work, we found that limiting levels of manganese in the growth medium impaired the recovery of DNA replication (Hutfilz *et al.* 2019). Based on these results, we proposed that H_2_O_2_ treatment oxidizes and inactivates some essential iron-dependent enzyme(s) and that manganese was needed to re-metallate these or alternative enzymes before genomic DNA replication can resume. These observations would be consistent with the results presented here and suggest that an oxidized ‘sensor’ protein is responsible for the observed inhibition rather than H_2_O_2_-induced lesions. The sensor could be a replication protein, or a protein that associates with the replisome to prevent DNA synthesis from progressing during oxygen stress. Alternatively, the sensor could be a repair protein(s) that is inactivated to prevent repair, thereby preventing replication from resuming. To examine this latter possibility, we compared the ability of cells to repair H_2_O_2_-induced lesions under varying manganese growth conditions. To this end, cultures were grown in defined medium supplemented with 200 µM MnCl_2_ or without additional metals, treated with H_2_O_2_, and monitored for the recovery of replication and repair of the H_2_O_2_-induced lesions as before. Although the absence of manganese reduced the ability of the cells to resume replication, it did not alter the rate or time that the H_2_O_2_-induced lesions were removed (Figure 4, A–C). Consistent with this, no replication fork processing intermediates were observed by 2D agarose gel analysis either in the presence or absence of manganese (Supplementary Figure S3). As a control, we also examined the rate of DNA synthesis and lesion removal following UV irradiation in the presence or absence of supplemented manganese. In contrast, to H_2_O_2_ treatment, the recovery of replication following UV irradiation occurred with similar kinetics in both the presence and absence of manganese supplementation. Repair of UV-induced lesions was similarly unaffected by the presence of manganese (Figure 4, D–F). The observation that manganese promotes the recovery of replication in H_2_O_2_-treated, but not UV-treated cultures, supports the idea that H_2_O_2_-induced replication inhibition results from an oxidized sensor that stalls replication, and is not due to disruption by DNA damage. Further, the results also demonstrate that lesion removal does not require manganese supplementation.

Taken together, the observations that replication recovers and continues in the presence of H_2_O_2_-induced DNA lesions, that RecF processing is not required for the H_2_O_2_-inhibited replication to resume, that processing intermediates at inhibited replication forks are not observed, and that the initial inhibition is not caused by a lack of repair, indicate that H_2_O_2_-induced DNA lesions do not disrupt replication *in vivo*. Instead, we infer that the initial inhibition of replication is likely to be caused by an iron-dependent replication protein or replication-associated protein that is oxidized and inactivates replication under these conditions.

## Discussion

The recovery and continuation of DNA synthesis suggests that the predominant lesions generated by H_2_O_2_ do not block replication progression *in vivo*. Early studies showed that thymine glycols, which comprise up to 30% of the H_2_O_2_-induced base damage, represent strong blocks to *E. coli* DNA polymerase I and phage T4 DNA polymerase *in vitro* (Ide *et al.* 1985; Rouet and Essigmann 1985; Clark and Beardsley 1986; Hayes and Leclerc 1986; Blakely *et al.* 1990), and it was often inferred that this would extend generally to other DNA polymerases. However, since these initial studies, several other polymerases have been examined and found to efficiently bypass this lesion, including many in humans (Fischhaber *et al.* 2002; Takata *et al.* 2006; Belousova *et al.* 2010; Yoon *et al.* 2010; Hogg *et al.* 2011; Makarova *et al.* 2018). Further, auxiliary proteins associated with the replisome, such as single-strand binding protein and processivity factors, can increase bypass efficiency in some cases (Maga *et al.* 2007; McCulloch *et al.* 2009). Finally, many of the other prominent lesions, such as 8-oxo-guanine, dihydrothymine, and uracil glycols are bypassed with fairly high frequencies *in vitro* (Purmal *et al.* 1994; Shibutani and Grollman 1994; Purmal *et al.* 1998; McCulloch *et al.* 2009). Taken together, these observations suggest that the replicative polymerases of *E. coli*, in the context of the full replisome, may bypass these lesions during genomic replication.

It is also possible that bypass frequencies at these oxidative lesions increase following the upregulation of manganese import that occurs after oxidative challenge (Kehres *et al.* 2002; Anjem *et al.* 2009). Consistent with this idea, we have previously shown that the recovery promoted by Mn is associated with an elevated level of mutagenesis that depends on both replicative and translesion polymerases (Hutfilz *et al.* 2019). Typical intracellular manganese concentrations in minimal medium can range from 15 to 150 µM when Mn transporters are fully induced (Anjem *et al.* 2009; Martin *et al.* 2015). The Mn-dependent mutagenesis may arise by reducing polymerase fidelity. Several studies have shown that manganese alters polymerase fidelity *in vitro*, including *E. coli* DNA polymerase I, human polymerases iota, Dpo4, and Primpol, and several polymerases used for PCR-mediated mutagenesis (Kunkel and Loeb 1979; Goodman *et al.* 1983; Beckman *et al.* 1985; Vaisman *et al.* 2005; Frank and Woodgate 2007; Tokarsky *et al.* 2017). If this phenomenon also extended to the replicative polymerase III in *E. coli*, it could explain why replication is initially inhibited, but then recovers in the absence of repair. However, the need for lesion bypass also predicts that replication should be disrupted by thymine glycols and depend on RecF processing for resumption, which is not observed.

Here we show that the inhibition and recovery of replication following H_2_O_2_-treatment is distinct from that following UV in several aspects. Following UV, the inhibition of replication results from disruption of the replisome by UV-induced pyrimidine dimers. In addition to the core proteins of the replication machinery, restoring replication following disruption *in vivo* requires processing of the fork by RecF and the repair of the lesions. In contrast, the inhibition of replication following H_2_O_2_ treatment appears to result from the inactivation of the replisome by an iron-sensitive protein. Restoring replication does not require lesion repair but is promoted by the presence of manganese. We did not detect any difference in the quantity of lesions present in DNA purified from cells grown in the presence or absence of manganese (Figure 4), arguing against the idea that the difference in recovery is due to a protective effect of manganese preventing lesion formation. However, we cannot exclude the possibility that different ratios or otherwise novel lesions are generated in these two conditions. Other differences that have been noted are that the increased mutation frequency after UV depends entirely on the UmuC polymerase, whereas after H_2_O_2_ the induced mutagenesis is more modest and involves both replicative and translesion DNA polymerases (Bagg *et al.* 1981; Imlay and Linn 1987; Napolitano *et al.* 2000; Hutfilz *et al.* 2019).

The results offer an explanation that may resolve a long standing paradox as to why oxidative glycosylase mutants are defective in repair, but not hypersensitive to H_2_O_2_ (Laspia and Wallace 1988; Asad *et al.* 1995; Saito *et al.* 1997; Spek *et al.* 2001; Schalow *et al.* 2011). However, several critical questions remain to be addressed as to the mechanism of cell death caused by oxygen free radicals. H_2_O_2_ induces a modest SOS response following H_2_O_2_ treatment (Imlay and Linn 1987; Goerlich *et al.* 1989; Gifford *et al.* 2000). Our current understanding suggests that the SOS response is typically induced in response to disrupted replication forks or double-strand breaks that subsequently form replication forks during repair (reviewed in (Walker 1984; Kogoma 1997; Hanawalt 2001; Crowley and Courcelle 2002)). The modest SOS induction observed could suggest that the replication fork still encounters some disruptive lesions either before inactivation occurs or due to incomplete inhibition of the forks. Early work has shown that cell lethality follows a curious bimodal pattern as the H_2_O_2_ concentration increases (Imlay and Linn 1986, 1987). ‘Mode 1’ killing occurs at concentrations below 2 mM H_2_O_2_ and appears to involve RecA and the SOS response. It could be these lower doses do not inactivate the replication machinery, allowing it to progress and be disrupted by the lesions, thereby inducing SOS. ‘Mode 2’ killing occurs at high concentrations, and may involve the inactivation of the replisome as we observed here. The H_2_O_2_ concentrations we used here would fall under the conditions of ‘mode 2’ killing. We did not explore the conditions associated with low dose or ‘mode 1’ killing, as the reduced number of lesions generated under these conditions would make their detection prohibitive with the assays employed here.

Alternatively, the modest SOS induction may suggest that oxidative damage induces double-strand breaks that are removed from the replication fork and arise elsewhere in the genome. This latter explanation would explain the lack of requirement for RecF which is not required for the repair of double-strand breaks and would also explain the H_2_O_2_ hypersensitivity of *recBC* mutants, which are defective in the repair of double-strand breaks (Willetts and Clark 1969; Willetts and Mount 1969; Horii and Clark 1973; Youngs and Bernstein 1973; Howard-Flanders 1975; Lloyd and Thomas 1983). Many iron-containing proteins bind DNA and James Imlay's group has proposed that these binding sites may be hotspots for DNA strand breaks due to Fenton chemistry generating oxygen free radicals at these loci (Imlay 2003; Djaman *et al.* 2004; Imlay 2014).

It is possible that the oxygen-sensitive pausing of replication represents an adaptive response to prevent genomic replication from occurring during periods of oxygen stress when potentially lethal or mutagenic damage is present. Pausing replication to allow more time for repair to occur has been demonstrated to be beneficial both for reducing mutations and increasing survival (Castellani *et al.* 1964; Ganesan and Smith 1969). This may also explain the increased resistance of *recF* mutants to H_2_O_2_ treatment, which are impaired in resuming replication after disruption (Figure 2A). In the absence of manganese, this inhibition can last for several hours, without compromising viability (Imlay and Linn 1986; Hutfilz *et al.* 2019).

There are other examples of oxygen sensors with the most notable example being OxyR, which following oxidation, transcriptionally upregulates genes associated with the oxidative stress response (Zheng *et al.* 1998). In the case of OxyR, activation/inactivation occurs through a reversible disulfide bond and does not involve iron or divalent metals. While this could also be the case with replication, the requirement for manganese to recover would argue that the sensor in this case is a metal-containing enzyme (Djaman *et al.* 2004; Anjem and Imlay 2012; Imlay 2014). Many mononuclear iron enzymes are inactivated by H_2_O_2_ treatment, several of which can be reactivated following re-metallation with manganese, which is insensitive to oxidation, to restore function (Anjem and Imlay 2012; Imlay 2014). The sensor inhibiting replication could be an essential component of the replisome itself or a secondary protein that upon oxidation, associates to inhibit replication. However, we use the term ‘sensor’ in this case in the broadest possible sense, since there are a large number of mechanistic possibilities one could envision that could regulate the inactivation and reactivation of the replisome following a strong oxidative challenge.

## Funding

This study was supported by National Science Foundation grant MCB1916625. 

##  

*Conflicts of interest*: None declared. 
